# Strain Elastography Ultrasound: An Overview with Emphasis on Breast Cancer Diagnosis

**DOI:** 10.3390/diagnostics3010117

**Published:** 2013-02-25

**Authors:** Jonathan F. Carlsen, Caroline Ewertsen, Lars Lönn, Michael B. Nielsen

**Affiliations:** Department of Radiology, Rigshospitalet, Copenhagen University Hospital, Blegdamsvej 9, Copenhagen OE DK-2100, Denmark; E-Mails: caroline.ewertsen@dadlnet.dk (C.E.); lönn.lars@gmail.com (L.L.); mbn@dadlnet.dk (M.B.N.)

**Keywords:** strain elastography, ultrasound, breast cancer, diagnostic accuracy

## Abstract

Strain elastography (SE), which estimates tissue strain, is an adjunct to the conventional ultrasound B-mode examination. We present a short introduction to SE and its clinical use. Furthermore, we present an overview of the 10 largest studies performed on the diagnostic accuracy of SE in breast cancer diagnostics. Eight of 10 studies presented data for both SE and B-mode imaging. Seven studies showed better specificity and accuracy for SE than for B-mode imaging in breast cancer diagnosis. Four studies showed an increase in specificity and accuracy when combining B-mode imaging with SE. The ways of combining B-mode imaging with SE in the diagnosis of breast cancer differed between the five studies. We believe that further studies are needed to establish an optimal algorithm for the combination of B-mode ultrasound and SE in breast cancer.

## 1. Introduction

Ultrasound elastography (UE) depicts the stiffness of tissues [1,2,3,4,5]. Just like manual palpation, elastography can distinguish a hard focal lesion from a soft lesion. Compared with manual palpation UE has the advantage of evaluating deeper lying lesions, and furthermore UE is semi-quantifiable. Several methods for semi-quantification exist today. One method uses a visual-scale score to evaluate the focal lesions; another uses strain-ratios and compares the strain of a region of interest in a focal lesion with the strain in the surrounding tissue. Elastography is available in most high-end ultrasound systems. This article presents an overview of strain elastography (SE) and its applications in breast cancer diagnostics.

## 2. Elastographic Technique

UE measures the strain of tissues [1,2,3,4,5]. The strain of a tissue is defined by the change in length during compression divided by the length before compression. The relationship between the compression, or the stress, and the strain is calculated in the Young’s modulus, E = stress/strain, which estimates the stiffness of a certain tissue [4]. Most elastography techniques measure the strain and not the Young’s modulus, and therefore a direct quantification is not possible. 

Strain elastography is available in most high-end ultrasound systems. SE measures axial displacement of tissue caused by mechanical stress in real-time. The stress is either applied externally with the transducer by the operator or by physiological shifts inside the patient. Transducer stress is applied by continuously compressing and decompressing the skin of the patient, a few millimeters of axial transducer movement being sufficient. 

**Figure 1:**
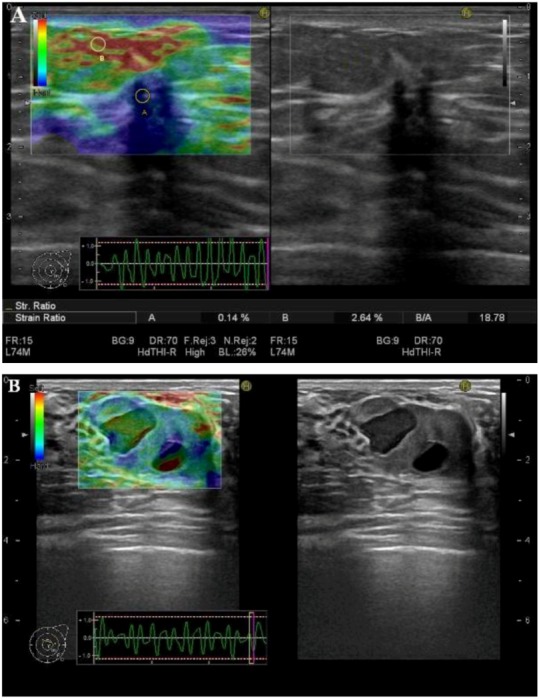
Co-registered Strain elastography (SE) (left) and ultrasound B-mode (right) images of (**A**) a hard (blue) malignant breast tumor (ductal carcinoma) and (**B**) a soft (green) benign breast neoplasm (fibroadenoma). The regions of interest (ROIs) placed in the tumor (A) and in the adjacent soft tissues (B) are used to calculate a strain-ratio (SR) (B/A) (right lower corner). The benign neoplasm (B) contains two hypoechoic areas, one of which yields a classic elastogram for a cyst with three color layers (blue-green-red) from top to bottom.

The elastogram is derived from data of the change of radio frequency signals before and after compression. The elastogram is displayed in a split-screen mode with the conventional B-mode image and the elastogram on the monitor (Figure 1). The elastogram may be displayed as a color-overlay on the B-mode picture. Most SE-systems display tissue stiffness in a continuum of colors from red to green to blue, designating soft (high strain), intermediate (equal strain) and hard (no strain) tissue. However, there is no color standard as yet, and some SE-systems have an inverse color scale of the others. As mentioned, SE measures the strain of tissues and not the Young’s modulus; thus, per definition the resulting elastogram is not quantifiable. There are several ways of providing a semi-quantifiable elastographic measure applicable in a clinical setting. These are especially useful when evaluating whether focal lesions are malignant or not.

Semi-quantifying elasticity was first described and used in the elastographic evaluation of breast tumors by Itoh *et al*. [6]. The visual scoring system compares the strain, *i.e*., the elastographic color of the lesion, with the strain of the surrounding tissue and can be applied in other anatomic areas as well [7,8,9,10,11].

The elastograms are divided into certain categories correlating to the likelihood of malignancy and range from benign (I), probably benign (II), uncertain (III), probably malignant (IV) to malignant (V) [6]. Another way of semi-quantifying the stiffness of a tissue is to use strain-ratios (SR) [12,13]. A SR-measurement compares the strain in two manually selected regions of interest (ROIs) on the elastograms. One ROI is placed in the focal lesion, and the reference ROI is placed in the surrounding normal tissue, preferably in the same depth as the lesion. The SR is automatically calculated by the elastography software and yields the fraction of the average strain in the reference area divided by the average strain in the lesion. The higher the SR, the higher the likelihood of malignancy. Cut-off values of the SR provide the highest diagnostic accuracy [13]. 

Elastographic examinations are highly operator dependent. Several studies have evaluated the interobserver variability of SE both in a phantom [14] and *in vivo* [7,8,9,10,11], but the results are not unanimous, since interobserver agreement ranges from high to low.

## 3. Clinical Applications

**Figure 2:**
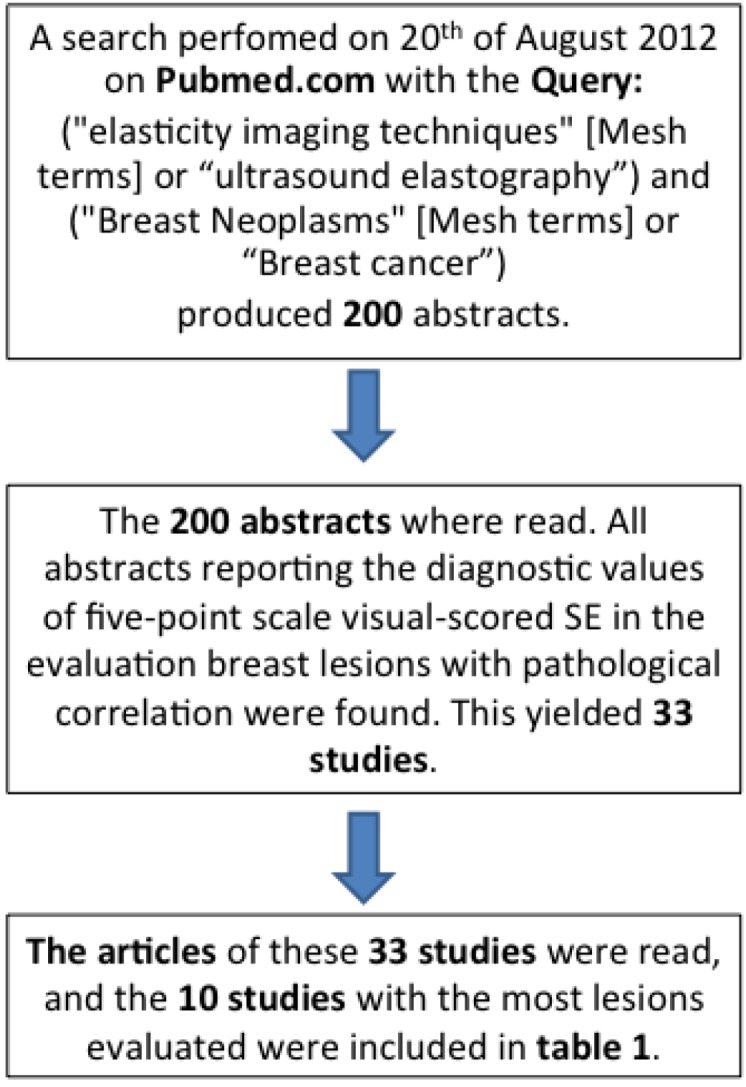
Schematic presentation of the search algorithm for the included studies.

SE has been applied in the ultrasound examinations of suspected breast cancer. The 10 largest published studies on visual scored elastography and breast cancer are listed in Table 1. These 10 studies were found on a search in Pubmed on the 20 August 2012. The search query contained the words “elasticity imaging techniques” (Mesh terms) or “ultrasound elastography”, and the words “Breast Neoplasms” (Mesh terms) or “Breast cancer” and yielded 200 abstracts. The abstracts were read by one author (JFC) and 33 articles met the inclusion criteria, *i.e*., reporting the diagnostic values of five-point scale visual-scored SE in the evaluation of breast lesions. Only studies on lesions without prior histological diagnosis were included. Of the 33 articles, 10 articles with the largest number of evaluated lesions were included in Table 1. The algorithm for the inclusion of these articles is illustrated in Figure 2. 

**Table 1:** Diagnostic values from the 10 largest published studies on semi-quantitative elastography and breast cancer. All listed studies used a five-point scale with a cut-off value between 3 and 4 for assessing malignancy. (*) Accuracy not reported in the paper, but calculated for this table. (¤) Cut-off values not reported in the paper, but reconstructed for this table. (#) Numbers are the diagnostic values of SR-measurements. (SFB) Scheduled for Biopsy.

**Study**	**Lesions biopsied (No. of patients)**	**Malignant/benign lesions (ratio)**	**Sensitivity**			**Specificity**			**Accuracy**			**Included lesions**
**(Publication year)**			**B-mode**	**SE**	**Combined**	**B-mode**	**SE**	**Combined**	**B-mode**	**SE**	**Combined**	
**Zhi *et al.*** [15]	401(370)	155/246 (0.63)	90.3	72.3	83.9	68.3	91.9	87.8	76.8	84.4	86.3	Lesions <2 cm
**Lee *et al.*** [16]	315(278)	48/267 (0.18)	95.8	35.4 (68.8)^#^	93.8	27.3	98.9^¤ ^(64.8)^#^	51.7	37.3^*^	89^*¤ ^(65.4)^#^	58.6^*^	Lesions <1 cm SFB
**Fu *et al.*** [17]	308(283)	104/204 (0.51)	82.7	66	97.1	87.7	88	71.9	86.0	81	80.5	All lesions <2 cm
**Wojcinski *et al.*** [18]	779(779)	360/419 (0.86)	95.0	81.2	na	76.1	89.0	na	84.9^*^	85.4^*^	na	Visible on B-mode, SFB
**Zhi *et al.*** [19]	559(437)	144/415 (0.35)	na	70.1 (92.4)^#^	na	na	93.0 (91.1)^#^	na	na	87.2 (91.4)^#^	na	BI-RADS 2-5
**Kumm *et al.*** [20]	310(288)	87/223 (0.39)	na	76 (79)^#^	na	na	81 (76)^#^	na	na	79 (77)^#^	na	Visible on B-mode, SFB
**Sohn *et al.*** [21]	281(267)	59/222 (0.27)	98.2	65.5	89.1	44.1	79	50.5	55.5^*^	76.2^*^	58.6^*^	Visible on B-mode, SFB
**Tan *et al.*** [22]	415(550)	119/431 (0.28)	86.6	78.0	Na	98.8	98.5	na	86.2	96.2	na	Visible on B-mode
**Zhi *et al.*** [23]	296(232)	87/209 (0.42)	71.2	70.1	89.7	73.2	95.7	85.7	72.6	95.0	93.9	Solid breast lesions
**Yi *et al.*** [24]	1,786(1,538)	263/1,523 (0.17)	98.5	34.2	Na	16.3	97.2	na	28.4*	87.9*	na	Non-palpable lesions

The inclusion criteria in the 10 selected studies differed somewhat, but all studies used the same five-point scoring system and a cut-off value between 3 and 4 for assessing the likelihood of malignancy, categories 1–3: benign and categories 4–5: malignant. The inclusion criteria for the lesions varied slightly, but all final diagnoses were made by pathological assessment of biopsies. Some studies only assessed small breast lesions (<2 cm [16,17] or <l cm [16]) as these are difficult to evaluate on B-mode imaging. 

### 3.1. Diagnostic Performance of SE

Eight of the 10 studies listed diagnostic values for both B-mode and SE [15,16,17,18,21,22,23,24]. All B-mode images in these eight studies were assessed by using ultrasound BI-RADS [25], a visual scoring system similar to the one used when assessing mammograms. The cut-off values for malignancy in B-mode imaging were only reported in four of eight studies [15,16,22,24]. Of these, three studies used a cut-off between 3 and 4 [15,22,24], and one study used further division of class 4 in to three subgroups, and a cut-off between 4a and 4b [16]. All eight studies comparing B-mode and SE-imaging showed a better sensitivity for B-mode than for SE (Table 1). Seven of these studies showed better specificity for SE than for B-mode imaging [15,16,17,18,21,23,24]. Accuracy was higher with elastography than B-mode imaging in seven of eight studies as well [15,16,18,21,22,23,24].

### 3.2. Combination of B-Mode and SE

Five of ten studies compared the diagnostic performance of B-mode-imaging with the diagnostic performance of the combination of B-mode and SE-imaging [15,16,17,21,23]. The two imaging techniques may be combined differently. The different methods used for combining B-mode ultrasound and SE are summarized in Table 2. Zhi *et al.* moderated the B-mode score according to SE-score, downgrading BI-RADS lesions 4 and 5 by one if the elasticity score was 1 or 2, and upgrading the BI-RADS lesions 1–3 to 4 if the elasticity score was 4 or 5 [15]. All other combinations were left unchanged. Lee *et al.* upgraded the B-mode score of all lesions with SE-score of 4 or 5 by one, downgraded lesions with SE-score of 1 by one and left lesions with SE-score of 2 and 3 unchanged [16]. Fu *et al.* categorized all lesions with either a malignant SE or B-mode score (or both) as malignant [17]. Sohn *et al.* left it to the sonographic evaluator’s judgment, whether or not to upgrade or downgrade the B-mode score after the SE-evaluation [21]. The combination was therefore done in a highly subjective and unformalized manner. The ways of combining B-mode and SE are not described in the fifth article [23]. 

Three of five studies reported a decrease in sensitivity when combining B-mode and SE, compared with B-mode-imaging [15,16,21]. The last two studies reported an increase in sensitivity when combining B-mode and SE [17,23]. Four of five studies reported an increase in specificity and accuracy when combining B-mode and SE [15,16,21,23].

A meta-analysis on the diagnostic value of visual scoring of SE in breast cancer diagnostics did not report the diagnostic values of B-mode-imaging or the combination of B-mode imaging and SE in the listed studies [26]. 

**Table 2:** Alteration of Breast Imaging Reporting and Data System (BI-RADS) score by the elasticity-score in the five studies, which reported diagnostic values for the combination of B-mode and SE-US. BI-RADS scores 1–3 usually designate a benign tumor, while BI-RADS 4–5 designate malignancy.

	**Elasticity-Score**				
	**1**	**2**	**3**	**4**	**5**
Zhi *et al.* [15]	Downgrade BI-RADS 4–5 by one		Unchanged	Upgrade BI-RADS 1–3 by one	
			BI-RADS score		
Lee *et al.* [16]	Downgrade all	Unchanged BI-RADS score		Upgrade all BI-RADS by one	
	BI-RADS by one				
Fu *et al.* [17]	Unchanged BI-RADS score		Upgrade BI-RADS 1–3 to malignant (≥4)		
Sohn. *et al.* [21]	Upgrade or downgrade of BI-RADS done unformalized by the evaluator				
Zhi *et al.* [23]	Method of combination of SE and B-mode US not described				

### 3.3. SR-Measurements

Two of the studies listed in Table 1 presented only the diagnostic values for SE [19,20]. These two studies and the study by Lee *et al*. [16] evaluated the diagnostic performance of visual scored SE with SR-measurements. The diagnostic values of SR-measurements are displayed in brackets. All three studies showed an increase in sensitivity and a decrease in specificity when using SR-measurements instead of visual scoring. Overall accuracy decreased in two of three studies.

Another meta-analysis highlighted a good diagnostic performance of SR-measurements in breast cancer diagnostics [27]. 

## 4. Conclusion

SE is widely available and easy to use in a clinical setting. The fact that SE is real-time and can be done bedside along with the B-mode examination makes the use of SE feasible in a lot of different anatomic areas. In breast cancer, SE has shown great potential and a good diagnostic performance in several studies. While sensitivity decreased in all eight studies comparing B-mode US with SE included in this review, specificity and accuracy increased in seven of eight studies. 

When comparing B-mode US with combined B-mode and SE, data are more controversial. The specificity and overall accuracy of the sonographic evaluation of breast lesions increased when combining B-mode- and SE-imaging in four of five studies presented in this article. 

Although SE has poor sensitivity, SE in combination with B-mode imaging shows better diagnostic performance. 

We suggest that elastography should be used as an adjunct to the clinical B-mode examination of suspected breast cancer. However, the algorithm used to combine the two diagnostic methods differed between the studies presented here. The best algorithm for the combination of B-mode and SE-imaging still needs to be established in future clinical studies. 
